# Improved services to enrollees into an HIV rural care and treatment center in Tanzania

**DOI:** 10.11604/pamj.2013.16.34.1642

**Published:** 2013-09-30

**Authors:** Khadija Said, Abdallah Mkopi, Suzanne Verver, Fred Lwilla, Gavin Churchyard, Seif Shekalaghe, Manuel Battegay, Klaus Reither

**Affiliations:** 1 Ifakara Health Institute, Bagamoyo, United Republic of Tanzania; 2 KNCV Tuberculosis Foundation, The Hague, The Netherlands and CINIMA, Academic Medical Centre Amsterdam, The Netherlands; 3 The Aurum Institute for Health Research, Johannesburg, South Africa; 4 Division of Infectious Diseases and Hospital Epidemiology-University Hospital Basel, Basel, Switzerland; 5 Swiss Tropical and Public Health Institute, Basel, Switzerland

**Keywords:** HIV treatment program, Sub-Saharan Africa, HIV, WHO clinical stage, CD4 cell count

## Abstract

Better quality of services is essential for the sustainability of HIV programs, in particular in rural Sub-Saharan Africa, to support the increasing number of individuals treated with combination antiretroviral therapy (cART). However, longitudinal data from rural care and treatment centers (CTC) are scarce. The objective was to assess trend in quality of care for HIV infected persons before start of combination antiretroviral therapy (pre-ART). A retrospective analysis of pre-ART registers and patient's files of 1950 patients enrolled in the Bagamoyo CTC in Tanzania between 2008 and 2010 analyzing was conducted; with parameters including year of enrollment, gender, age, CD4 cell count and WHO clinical stage at time enrollment. We noted a significant increase by 20% of total patients who had CD4 cell count performed from 69% (n=457) in 2008, 83% (n=493) 2009 to 89% (n=616) 2010 (X^2^= 87.014, P^2^= 14.945, P^2^= 85.028, P^3^. Efforts must be undertaken for more HIV testing and timely referral of HIV-infected patients to CTC.

## Introduction

HIV is a major public health challenge worldwide, in particular in Sub-Saharan Africa [1]. Scaling up access to HIV prevention, treatment and care are the main achievements and goals of the WHO, UNAIDS and other organizations. Scaling up is particularly significant in sub-Saharan Africa where more than 5 million individuals are treated with cART as reported by the WHO [2].

Many HIV-programs had to be installed very rapidly with no adequate preparation, hence compromise programs as a result information collected may not be of high quality. For example, a study from 15 resource limited countries showed that quality of the data collected in treatment programs are often unsatisfactory mainly because of insufficient or in adequately trained staffs [3]. Along this, still many HIV-infected individuals are referred to Care and Treatment Centers (CTC) late in their HIV disease [4] and that data on their admission characteristics especially from rural CTC are scarce.

Well planned CTC programs will speed up reduce new HIV infections. This is stated in the recent report from UNAIDS, that quality programs should be increased and sustainably reach different parts.[2, 5]. Here, we analyzed retrospectively baseline information collected in the ongoing pre-ART registers and patient's files of patients enrolled between 2008 and 2010 to the Bagamoyo CTC. We investigated the trend of quality of services offered to enrollees in Bagamoyo CTC by looking at documentation of various baseline parameters recommended by the National guideline including demographic information, year of enrollment, CD4 cell count and World Health Organization (WHO) clinical stage.

## Methods

### Study site

Data were collected from the Bagamoyo CTC. This clinic was established in 2005 and serves the entire district. The clinic is run jointly by the Ministry of Health and the International Centre for AIDS care and the treatment program of the Columbia University, USA. Care and treatment at this clinic follows Tanzanian National HIV care and treatment guidelines of 2008

### Study population

Data were collected from all patients (n=1950) who attended Bagamoyo CTC from January 2008 to December 2010. Patients included those who primarily attended Bagamoyo CTC either for treatment or voluntary counseling and testing for HIV as well as those referred from various sections within Bagamoyo hospital (Reproductive and child health, Outpatient, Inpatients, Home based care) and people living with HIV/AIDS. 30 patients were excluded from analysis because they were either referred from other CTCs or their records missed demographic information such as gender, age or date of birth because they were not documented at the time of enrolment

### Data Collection and analysis

Data have been retrospectively collected from the pre-ART register and patient files (2008-2010) where all tested HIV positive individuals and those that are referred from other CTC are documented. From the pre-ART register, we collected baseline information which include demographic information, laboratory investigations taken prior to starting combination antiretroviral therapy (cART)and also secondary parameter such as WHO clinical staging.

### Ethical Consideration

Before data collection, protocol was submitted for ethical consideration. Approval to collect data was sought from Ifakara Health Institute Institutional Review Board (IHI-IRB) and the National Institute for Medical Research-Medical Research Coordinating Committee (NIMR-MRCC) of Tanzania and permission from the local authorities in Bagamoyo District.

### Statistical analysis

Data was analyzed using Stata (version 11, College Station, Texas, USA). Frequency distribution and summary statistics were performed appropriately. Chi square for trend was calculated to determine trend over years.

## Results

### General Characteristics of the Patients

Majority of participants were aged between 20-40 years 60% (1179/1950) with median age of 35 years (IQR 28-43). Females were many 67% (1297/1950) compared to males. The number of patients enrolled to the CTC was relatively stable in each year (Table 1) and majority of them were self referral 65% (1270/1950).

**Table 1:** Baseline characteristics of participants enrolled at Bagamoyo CTC

Characteristic	Total	Year of enrollment (N = 1950)		
		2008	2009	2010
		n=663	n=596	n=691
**Gender n (%)**				
Female	1297 (67)	440(66)	406(68)	451(65)
Male	653 (34)	223(34)	190(32)	240(35)
**Age (years)**				
Median		35	34	35
Interquartile range		28-42	28-42	28-44
**CD4+ cells/ mm^3^**				
Median		229	258.5	246.5
Interquartile range		69-458	91-475	106-437

The median CD4 cell count was 244cells/mm^3^ (IQR 86-456) and 34% (670/1950) were diagnosed with CD4 cell count below 200cells/mm^3^ (Table 2). Overall, there was 20% increase in total patients who had CD4 cell count performed from 69% (n=457) in 2008 (test performed in another laboratory in the region), 83% (n=493) 2009 (test performed in Bagamoyo CTC laboratory) to 89% (n=616) 2010 (test performed in Bagamoyo CTC laboratory) (X^2^=87.014, P^3^ were females. Interestingly, 20% (384/1950) of patients had no CD4 cell count measured and no reasons documented in their files, however the District AIDS coordinator and CTC in-charge mentioned lack of CD4 cell count machine in 2008, supplies mostly blood tubes and reagents as well as personnel to be the main reasons. We also found a decrease in patients with missing WHO clinical stage documented from 20% (n=135) in 2008, 19% (n=113) in 2009 to 7% (n=49) in 2010.

**Table 2:** CD4 cell count and WHO clinical stage of participants enrolled at Bagamoyo CTC. January 2008-December 2010 (N = 1950)

Indicator	Total	2008	2009	2010	p-value*
CD4 cell count n(%)		n=663	n=596	n=691	
≤200cells/mm^3^	670 (34)	208 (31)	203 (34)	259 (37)	0.0179
200-350cells/mm^3^	337 (17)	99 (15)	100 (17)	138 (20)	0.0141
>350cells/mm^3^	559 (29)	150 (23)	190 (32)	219 (32)	0.0003
Not done	384 (20)	206 (31)	103 (17)	75 (11)	<0.0001
**WHO clinical Stage n (%)**					
Stage 1	471 (24)	103 (16)	113 (19)	255 (37)	<0.0001
Stage 2	409 (21)	105 (16)	101 (17)	203 (29)	<0.0001
Stage 3	551 (28)	233 (35)	179 (30)	139 (20)	<0.0001
Stage 4	222 (11)	87 (13)	90 (15)	45 (7)	0.0001
Missing	297 (15)	135 (20)	113 (19)	49 (7)	----

* p-value: chi square for linear trend

Clinically 222 (11%) patients presented with advanced HIV disease i.e. WHO clinical stage 4 disease. There was a decrease noted from 87 (13%) in 2008 to 45 (7%) in 2010 (X^2^ =14.945, P^2^=85.028, P

## Discussion

In this study we investigated 1950 patients who had been enrolled into the CTC. Our results showed that: a) the number of missing data for CD4 count was decreasing over three years, b) the percentage of late presentation remained stable at approximately one third of patients, and c) enrollees had more frequently presented without clinical signs of HIV.

Hence, the quality of services, in particular the reliable CD4 count measurement during enrolment of patients in CTC has improved. This maybe well explained by presence of a CD4 cell count machine in the district laboratory since late 2008 indicating the importance of well equipped laboratories also in more remote rural areas. WHO recommends laboratory monitoring of both cART naïve and cART experienced patients whenever possible to improve the care of HIV-infected individuals. In line, viral load could not be measured because at this CTC there are no equipments to measure viral load.

Our data indicates that also after three years late presentation of HIV is a problem in the region as reported elsewhere [6]. Nevertheless, we noted an increase of patients in early clinical stages of their HIV infection. This may indicate that once symptoms have developed patients seek help. Still, efforts should be made for more voluntary counseling and testing to ensure earlier diagnosis of HIV in individuals with no or mild symptoms [7].

Interestingly, despite cART scaling up in Tanzania the overall number of patients enrolled from 2008 to 2010 was stable. This is likely due to the fact that other CTC, namely in Chalinze, Kiwangwa, Lugoba, Mbwewe, Miono and Msata are now also operating in the district.

Noteworthy, more patients were females consistent with a study by Somi et al with a female to male ratio of 2:1, also reported from Tanzania [8]. This may be due to more entry points women have to CTC services such as via antenatal clinics. The importance of voluntary testing and counseling is confirmed by our study as the majority, i.e. 65% came after voluntarily testing to the CTC [9].

With a treatment threshold below 200 cells/mm^3^ about 34% of patients were eligible for cART which is a lower percentage as found in South Africa with 54% of patients eligible for ART [10] Possibly lacking CD4 cell counts in the CTC in Bagamoyo led to underestimation of eligibility for cART. Taking the new WHO 2010 ART guidelines into account, approximately half of patients in our study would have to start cART.

As a limitation we would like to mention that we could analyze only a few parameters restricted to the first three months of enrolment. Nevertheless, our results indicate a slight progress of quality of services as assessed by documentation of clinical WHO stage and by reliability of CD4 cell count measurements over three years.

## Conclusion

These findings indicate that services offered to HIV infected persons pre-ART has progressively improved at the Bagamoyo CTC between 2008 and 2010. However, one out of three patients has still CD4 cell count below 200cells/mm^3^ at the first visit. More frequent HIV testing and timely referral of HIV-infected patients to CTC would further improve quality of care for HIV infected persons.
